# Biosynthesis of Conjugate Vaccines Using an O-Linked Glycosylation System

**DOI:** 10.1128/mBio.00443-16

**Published:** 2016-04-26

**Authors:** Chao Pan, Peng Sun, Bo Liu, Haoyu Liang, Zhehui Peng, Yan Dong, Dongshu Wang, Xiankai Liu, Bin Wang, Ming Zeng, Jun Wu, Li Zhu, Hengliang Wang

**Affiliations:** a State Key Laboratory of Pathogen and Biosecurity, Beijing Institute of Biotechnology, Fengtai, Beijing, China; b National Institutes for Food and Drug Control, Beijing, China

## Abstract

Conjugate vaccines are known to be one of the most effective and safest types of vaccines against bacterial pathogens. Previously, vaccine biosynthesis has been performed by using N-linked glycosylation systems. However, the structural specificity of these systems for sugar substrates has hindered their application. Here, we report a novel protein glycosylation system (O-linked glycosylation via *Neisseria meningitidis*) that can transfer virtually any glycan to produce a conjugate vaccine. We successfully established this system in *Shigella* spp., avoiding the construction of an expression vector for polysaccharide synthesis. We further found that different protein substrates can be glycosylated using this system and that the O-linked glycosylation system can also effectively function in other Gram-negative bacteria, including some strains whose polysaccharide structure was not suitable for conjugation using the N-linked glycosylation system. The results from a series of animal experiments show that the conjugate vaccine produced by this O-linked glycosylation system offered a potentially protective antibody response. Furthermore, we elucidated and optimized the recognition motif, named MOOR, for the *O*-glycosyltransferase PglL. Finally, we demonstrated that the fusion of other peptides recognized by major histocompatibility complex class II around MOOR had no adverse effects on substrate glycosylation, suggesting that this optimized system will be useful for future vaccine development. Our results expand the glycoengineering toolbox and provide a simpler and more robust strategy for producing bioconjugate vaccines against a variety of pathogens.

## IMPORTANCE

Recently, the rapid development of synthetic biology has allowed bioconjugate vaccines with N-linked protein glycosylation to become a reality. However, the difficulty of reestablishing the exogenous polysaccharide synthetic pathway in *Escherichia coli* hinders their application. Here, we show that an O-linked protein glycosylation system from *Neisseria meningitidis*, which has a lower structure specificity for sugar substrates, could be engineered directly in attenuated pathogens to produce effective conjugate vaccines. To facilitate the further design of next-generation bioconjugate vaccines, we optimized a novel short motif consisting of 8 amino acids that is sufficient for glycosylation. Our results expand the application potential of O-linked protein glycosylation and demonstrate a simpler and more robust strategy for producing bioconjugate vaccines against different pathogens. In the future, bacterial antigenic polysaccharides could be attached to major histocompatibility complex binding peptides to improve immunological memory or attached to protein subunit vaccine candidates to provide double immune stimulation.

## INTRODUCTION

Conjugate vaccines, created by covalently attaching a bacterial antigenic polysaccharide to a carrier protein, can activate a T-cell-dependent immune response (1–3) and are one of the most successful weapons for the prevention of infectious disease, especially in infants and older people (4). To date, many types of conjugate vaccines against *Haemophilus influenzae* type b, *Streptococcus pneumoniae*, *Neisseria meningitidis*, and *Salmonella enterica* serovar Typhi, among others, have been licensed and have superb safety and efficacy, especially the seven-valent pneumococcal conjugate vaccine Prevnar (PCV7) for infant immunization, which was licensed in the United States in 2000. By 2004, the rates of all-cause pneumonia admission and of hospitalizations for pneumococcal meningitis decreased by 39% and 66%, respectively, in children younger than 2 years (5, 6). To our knowledge, all of the licensed conjugate vaccines, such as Hiberix, Menveo, Prevnar, and Synflorix, are created by chemical methods. However, such methods involve a multistep strategy that includes several purification processes, which greatly increases their cost and thereby limits the market for these vaccines in developing countries.

Biosynthesis of polysaccharide conjugate vaccine production is evolving. In the last two decades, glycosylation pathways have been discovered in bacteria. The two best understood of these are the N-linked glycosylation system discovered in *Campylobacter jejuni* (7–9) and the O-linked glycosylation system found in *Neisseria* species (10, 11). In these two systems, the bacterial polysaccharide is transferred from an undecaprenyl pyrophosphate (UndPP) carrier onto the protein acceptor. This process is similar to the Wzy-dependent O-antigen biosynthetic system that transfers glycans onto the lipid A core (12, 13). Further, the two glycosyltransferases, PglB from *C. jejuni* (N linked) and PglL from *Neisseria* (O linked), also can be functionally transferred into *Escherichia coli* alone and are capable of mediating long glycan transfer (8, 11). PglB, which is homologous to the Stt3 component of the oligosaccharyltransferase (OTase) complex in eukaryotic cells (14), was the first to be used to produce conjugate vaccines because its glycosylation sequon was clear, a conserved pentapeptide motif, D/E-X_1_-N-X_2_-S/T (where X_1_ and X_2_ are any residues except proline), unlike the tripeptide motif NXS/T (where X can be any amino acid except proline) that is present in eukaryotic cells (15). This motif can be fused to a carrier protein to generate a glycoprotein (16). A promising bioconjugate against *Shigella dysenteriae* produced by genetically modified *E. coli* using this N-linked glycosylation system has been developed by the company GlycoVaxyn AG and was recently tested in a phase 1 clinical trial (16, 17). However, PglB works only if the sugar substrate contains an acetamido group at position C-2 of the reducing end and does not possess a β1-4 linkage between the first two sugars (18, 19). Polysaccharides in some bacteria, such as *S. pneumoniae*, *Streptococcus suis*, and *Salmonella*, lack appropriate reducing ends (20–23) and cannot be used in this system. Additionally, in many other bacteria, the gene cluster associated with the assembly of antigenic polysaccharides is still unknown, making the expression of exogenous polysaccharides difficult.

In *Neisseria meningitidis*, the glycosyltransferase PglL has been shown to be responsible for the attachment of the UndPP-glycan to pilin (PilE), which generates a glycoprotein, and one of the glycosylation sites on PilE is Ser63 (24, 25). Previous work has demonstrated that PglL can transfer virtually any glycan to pilin *in vivo* (26), and the only requirement for this process is that the glycan must be carried by a lipid carrier, UndPP. PglL, therefore, has more potential applications than PglB. However, unlike PglB, the structural determinants intrinsic to PglL have been difficult to characterize (27), and this has prevented the O-linked system from being used to produce conjugate vaccines. In *N. meningitidis*, at least seven proteins can be glycosylated by PglL (28), but as in eukaryotes, the sequons around glycosylation sites are of low complexity and do not follow a single pattern (29). The peptide-glycan combination is important for antigen recognition and presentation (3), and the next generation of conjugate vaccines might be designed based on the location of this sugar-peptide glycosidic bond (30). In cases where the recognition sequence for O-link glycosylation is known, the bacterial antigenic polysaccharide can be attached to major histocompatibility complex (MHC) binding peptides to further enhance vaccine efficacy.

The goal of this study was to develop a novel synthetic biology strategy to produce bioconjugate vaccines, providing an alternative to conventional N-glycosylation biological methods. Here, we report a novel protein glycosylation system that uses O-linked glycosylation from *N. meningitidis* to produce a vaccine. We show that glycoproteins with different carriers can be achieved by engineering directly in attenuated pathogens, and this type of bioconjugate can evoke a protective and specific immune response. Further, we elucidated and optimized the recognition motif, named MOOR, for the *O*-glycosyltransferase PglL, and then we generated a novel bioconjugate by fusing it to peptides that are recognized by MHC class II (MHC-II) to improve its presentation ability. Our results demonstrate a simpler and more robust strategy for producing bioconjugate vaccines against a variety of pathogens, expanding the application potential of O-linked protein glycosylation and, by site optimization, enabling the use of O-linked glycosylation to produce the next generation of conjugate vaccines.

## RESULTS

### Establishment of an O-linked glycosylation system in the attenuated *Shigella flexneri* strain 301DWP.

**FIG 1:**
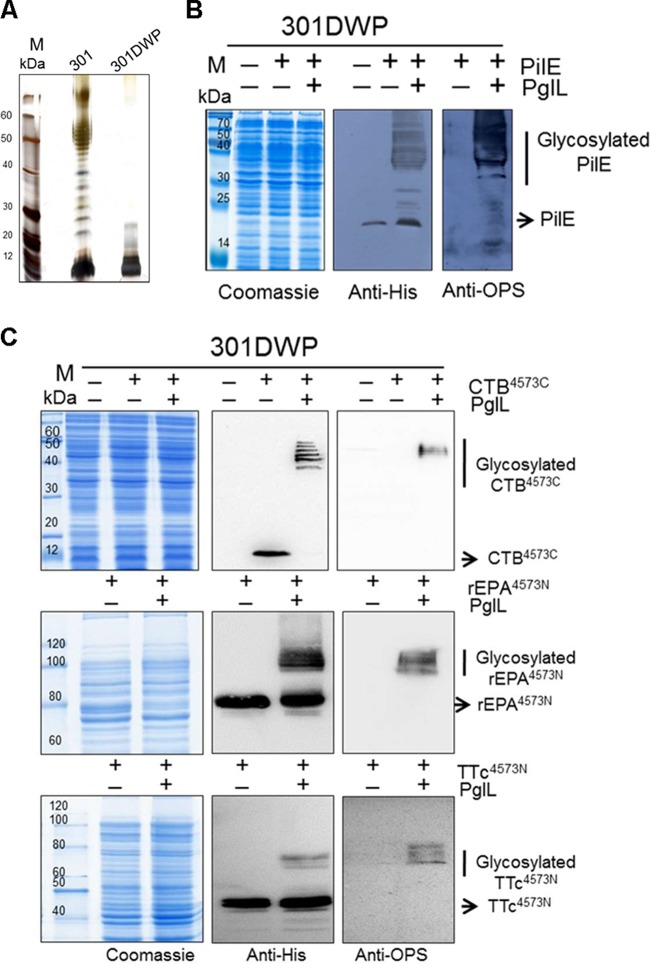
Establishment of an O-linked glycosylation system in the attenuated *S. flexneri* strain 301DWP. (A) Silver staining of LPS from *S. flexneri* 2a strains 301 and 301DWP. (B and C) Coomassie blue staining and Western blot assays to analyze the glycosylation of the natural substrate protein, PilE (B), or of recombinant substrates, CTB protein fused with the PilE S^45^-K^73^ fragment (4573) at the C terminus or recombinant rEPA or TTc proteins fused with 4573 at the N terminus (C), when each was coexpressed with the corresponding O-linked glycosyltransferase PglL.

*Escherichia coli* strains, such as CLM24 (16), are the bacteria that have been most commonly employed as host strains in biomethods to produce conjugate vaccines, and multiple plasmids are required to express carrier proteins, PglB, and the glycan gene cluster together in these strains. To make the production of conjugate vaccines simpler, we adopted a new strategy that involves using the attenuated pathogen as the host strain to produce glycoproteins. Here, we used *S. flexneri* 2a strain 301 as the host, and because the O-antigen ligase gene *waaL* from this strain is responsible for the transformation of *O*-polysaccharide (OPS) from UndPP to the lipid A core, we used the λ-red recombination system (31) to delete this gene (see Text S1 in the supplemental material), resulting in a lipopolysaccharide (LPS)-deficient attenuated strain. For biosafety, this strain was further attenuated by curing the virulence plasmid using plasmid incompatibility, and the resulting strain was named 301DWP (see Fig. S1A in the supplemental material). To confirm the phenotype of this mutant, the LPS of each strain was analyzed by silver staining, and no LPS ladder was detected in strain 301DWP (Fig. 1A). To evaluate virulence, Sereny test and plaque formation experiments were performed (see also Text S1 in the supplemental material). The results show that compared with the wild-type strain, strain 301DWP did not cause inflammation of the cornea (see Fig. S1B in the supplemental material), and plaques were hardly detectable (see Fig. S1C in the supplemental material). These results indicate that the virulence of *S. flexneri* 2a strain 301 was mostly determined by the virulence plasmid and the surface glycan and that the 301DWP strain, which lacks these, is not virulent.

PilE, a structural component of type IV pilin, is the natural substrate of PglL that catalyzes the O-linked glycosylation reaction in *Neisseria*. To verify that this system could work effectively in other pathogens, such as *S. flexneri* 2a strain 301DWP, plasmids expressing an inducible PglL (pET-pglL) and an inducible substrate protein PilE (pMM-pilE) were introduced into strain 301DWP. The Western blot results show the typical ladders caused by different sugar repeat units for the isopropyl-β-d-thiogalactopyranoside (IPTG)-induced 301DWP strain coexpressing PilE and PglL (Fig. 1B). The molecular mass was about 30 kDa for OPS chains with a modal length of 11 to 17 repeated units controlled by the chromosomally encoded Wzz protein (32, 33).

PilE can be glycosylated at Ser63 (24, 25). To determine whether or not this O-linked glycosyltransferase could attach an antigenic polysaccharide to widely used commercial carrier proteins, such as recombinant *Pseudomonas aeruginosa* exotoxin A (rEPA), tetanus toxin C fragment (TTc), and cholera toxin b subunit (CTB), we added a peptide fragment (^45^SAVTEYYLNHGEWPGNNTSAGVATSSEIK^73^) surrounding S^63^ (underlined) onto the N termini of rEPA and TTc and onto the C terminus of CTB (pMM-CTB^4573C^, pMM-rEPA^4573N^, and pMM-TTc^4573N^) (Table 1). rEPA and TTc have been widely used for vaccine research, and some conjugate vaccines using these carriers, such as ActHib, Hiberix, and StaphVAX, have been licensed or are under investigation in a phase 3 clinical trial. CTB also has been widely used as carrier because of its adjuvant activity (34). The Western blot results show that, when the corresponding plasmids were transformed into the 301DWP strain harboring the pET-pglL plasmid, our recombinant carrier proteins were efficiently glycosylated following induction with IPTG (Fig. 1C). Notably, the glycosylation efficacy was highest when CTB^4573C^ was used as a carrier, in which case almost all of the substrate proteins were glycosylated (Fig. 1C).

**TABLE 1:** Main plasmids used in this study

Plasmid	Characteristic^a^	Source
pKD46	Used for λ-red recombination, araC-ParaB, Ap*^r^*	Laboratory stock
pET-kan	Carries a kanamycin resistance gene flanked by FRT sites	Laboratory stock
pCP20	Used for the removal of kanamycin resistance gene	Laboratory stock
pMM-pilE	Encodes 6×His-tagged PilE under control of Tac promoter, Amp^r^	This work
pET-pglL	Encodes PglL under control of Tac promoter, Kan^r^	This work
pMM-CTB^4573C^	Encodes 6×His-tagged CTB and fuse DsbA signal peptide at N terminus and PilE4573 fragment at C terminus under control of Tac promoter, Amp^r^	This work
pMM-rEPA^4573N^	Encodes 6×His-tagged rEPA and fuse DsbA signal peptide at N terminus and PilE4573 fragment at N terminus under control of Tac promoter, Amp^r^	This work
pMM-TTc^4573N^	Encodes 6×His-tagged TTc and fuse DsbA signal peptide at N terminus and PilE4573 fragment at N terminus under control of Tac promoter, Amp^r^	This work
pET-pglL-CTB^4573C^	Encodes PglL and 6×His-tagged CTB from pMM-CTB^4573C^, both of them under control of Tac promoter, Kan^r^	This work
pET-pglL-rEPA^4573N^	Encodes PglL and 6×His-tagged rEPA from pMM-rEPA^4573N^, both of them under control of Tac promoter, Kan^r^	This work

a Abbreviations: araC, 1-β-d-arabinofuranosylcytosine; FRT, FLP recombination target.

### Application potential of the O-linked glycosylation system.

**FIG 2:**
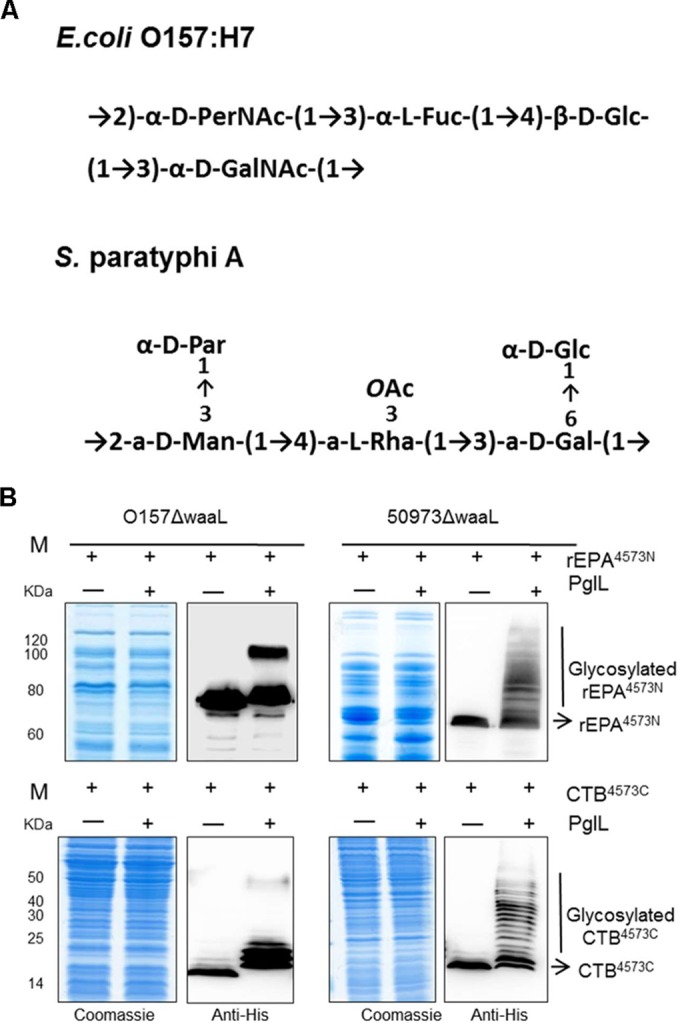
O-linked glycosylation in different pathogens. (A) The repeating unit structures of the OPS of *E. coli* O157:H7 (top) (35) and *Salmonella* serovar Paratyphi A (bottom) (36). (B) The O-linked glycosylation system was introduced into *E. coli* O157:H7 or *Salmonella* serovar Paratyphi A strain CMCC 50973, each of which lacked *waaL*, to test the universality of this system. Coomassie blue staining and Western blot assays were used to analyze the glycosylation of the substrate proteins rEPA^4573N^ (top) and CTB^4573C^ (bottom) expressed alone or coexpressed with PglL.

To determine if this system could be applied to other pathogens, we used *E. coli* O157:H7, a serious gut pathogen, and *Salmonella enterica* serovar Paratyphi A strain CMCC 50973, whose OPS cannot be recognized by PglB because the sugar substrate does not contain an acetamido group at position C-2 of the reducing end. The structures of their repeating OPS units are shown in Fig. 2A (35, 36). The *waaL* gene was deleted in each of these strains using the method described above, and the plasmids pET-pglL-rEPA^4573N^ and pET-pglL-CTB^4573C^ (Table 1) were then introduced into these mutants. The Western blot results show that the carrier proteins rEPA^4573N^ and CTB^4573C^ were efficiently glycosylated in both strains (Fig. 2B), indicating that this modified O-linked glycosylation system might be adopted by many other Gram-negative pathogens. Furthermore, the *E. coli* strain CLM24, which is commonly used in glycosylation research, was also compatible with our O-linked glycosylation system (see Fig. S2 in the supplemental material). That is, all reported applications of N-linked glycosylation could be theoretically imitated in our O-linked glycosylation system. Furthermore, some cases that could not be completed in the PglB system might be achieved using the PglL system.

### Purification of the glycoproteins.

**FIG 3:**
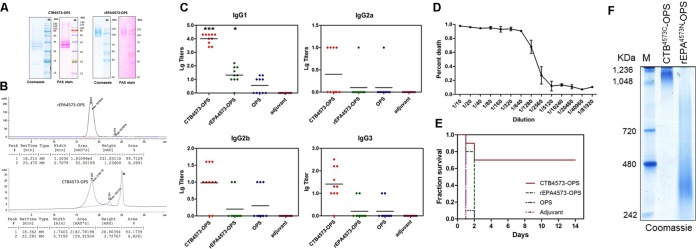
Evaluation of the glycoproteins isolated from animal experiments. (A) Purified CTB^4573C^-OPS and rEPA^4573N^-OPS were separated by 12% and 10% SDS-PAGE, respectively. The proteins were then stained by Coomassie blue, and the OPSs were visualized by PAS staining. (B) For analysis with SEC-HPLC (TSK G4000SWXL, diameter [φ], 7.8 × 300 mm), 25 µl of purified glycoproteins was added, and the mobile phase consisted of 50 mM phosphate buffer and 0.9% NaCl. (C) The IgG subtype titers (IgG1, IgG2a, IgG2b, and IgG3) against the LPS from *S. flexneri* 2a strain 301 were measured in the serum of BALB/c mice immunized with CTB^4573C^-OPS, rEPA^4573N^-OPS, OPS, or adjuvant alone. Data are shown as points, and the lines indicate the means. Compared with the OPS group by *t* test, *t* was 15.4 for CTB^4573C^-OPS and *t* was 2.76 for rEPA^4573N^-OPS (*, *P* < 0.05; ***, *P* < 0.0001). (D) Complement bactericidal activity in different dilutions of serum from the mice vaccinated with CTB^4573C^-OPS. The error bars indicate the range for the percentage of deaths. (E) Mice that had been vaccinated as described for panel C were infected intraperitoneally with approximately 3 × 10^7^ CFU/mouse of *S. flexneri* 2a strain 301, and their survival was monitored. (F) BN-PAGE analysis of CTB^4573C^-OPS and rEPA^4573N^-OPS.

To detect the immunogenicity of our bioconjugates, purification was performed to obtain glycosylated rEPA^4573N^ and CTB^4573C^, and the levels of their production were detected by Coomassie blue and periodic acid-Schiff (PAS) staining (Pierce glycoprotein staining kit; Thermo Scientific) (Fig. 3A). Their purities were further detected as 99.7% and 93.2%, respectively, by size exclusion high-performance liquid chromatography (SEC-HPLC) (Fig. 3B), and these purities were sufficient for our subsequent experiments. Next, protein and glycan concentrations were measured via the micro-bicinchoninic acid (micro-BCA) method (MicroBCA protein assay kit; Thermo Scientific) and anthrone-sulfuric acid method (see also Text S1 in the supplemental material), and the results show that the concentrations of rEPA4573-OPS and CTB4573-OPS were 673 µg/ml and 965 µg/ml, respectively. Additionally, approximately 9 mg and 6 mg of high-purity rEPA^4573N^-OPS and CTB^4573C^-OPS, respectively, could be achieved per liter of culture broth at the experimental stage. Furthermore, it is likely that the glycoconjugate yields could be increased by optimizing the medium and culture conditions in medium-scale experiments.

### Immune evaluation of the glycoproteins.

The glycoproteins (rEPA^4573N^-OPS and CTB^4573C^-OPS) and OPS were used, along with 10% Al(OH)_3_ adjuvant (General Chemical), to immunize BALB/c mice (5 weeks old, 10 per group; 2.5 µg polysaccharide/mouse) subcutaneously on days 1, 15, and 29, with the adjuvant-only group as a control. Serum was harvested on day 39 by tail snip, and the subtype concentrations of antibodies to the LPS of *S. flexneri* 2a strain 301 were evaluated by enzyme-linked immunosorbent assays (ELISAs) using peroxidase-conjugated goat anti-mouse IgG1, IgG2a, IgG2b, and IgG3 as the secondary antibodies. Among these subtypes, the titer of IgG1 induced by the CTB^4573C^-OPS group was dramatically higher than that of the rEPA^4573N^-OPS group, although the titers of both groups were higher than those of the OPS and control groups (Fig. 3C).

Additionally, because IgG1 can activate the classical complement pathway, complement bactericidal activity assays were performed to further investigate the immune effects induced by CTB^4573C^-OPS. When the antigens and antibodies were combined, the classical complement pathway was activated, and CTB^4573C^-OPS serum induced a high level of complement bactericidal activity. The sterilization rate reached 90% at serum dilutions of less than 640 (Fig. 3D). Further, to confirm the protective effects of this vaccine, a survival experiment was performed. By intraperitoneally infecting mice with approximately 3 × 10^7^ CFU/mouse of *S. flexneri* 2a strain 301 on day 43 (14 days after the last vaccine or control injection), we observed that deaths mainly occurred in the first few days, and the protection rate for the CTB^4573C^-OPS group was 70%, which is higher than those of the other vaccine candidates or control substances (Fig. 3E). Taken together, these experiments show that vaccination with CTB^4573C^-OPS is potentially protective.

CTB exists in a pentameric form (37), whereas rEPA is monomeric in nature. We hypothesized that CTB^4573C^-OPS performed better as a vaccine than rEPA^4573N^-OPS because purified CTB^4573C^-OPS also formed a pentamer. To test this idea, blue native-polyacrylamide gel electrophoresis (BN-PAGE) was performed using a Ready-Gel with a linear 4 to 15% gradient (catalog no. 1611104; Bio-Rad) according to the manufacturer’s instructions, and the results show that the molecular mass of the polymer was more than 1,000 kDa, while the molecular mass of the CTB^4573C^-OPS monomer was only about 40 kDa (Fig. 3F). Interestingly, the measured molecular masses of both CTB^4573C^-OPS and rEPA^4573N^-OPS were higher than predicted, possibly because of the poor electrophoretic mobility of the glycan or the branching structure of the glycoproteins (Fig. 3F). In N-glycosylation studies, rEPA has been successfully used as a carrier protein to produce conjugate vaccines (16). However, our results show that CTB is a more effective carrier protein than rEPA.

### The original glycosylation sequence could be truncated to 10 amino acids.

All of our above data support the use of the modified O-linked glycosylation system for the development of bacterial bioconjugate vaccines. However, two significant problems still hindered its application: the O-glycosylation site was not clear, and the amino acids conserved within the glycosylation motif remained to be determined. In our previous work, the peptide recognized by PglL was 29 amino acids in length, which is longer than the peptides that can be presented by MHC-II molecules (generally 15 to 24 amino acids long). Because the efficacy of these types of bioconjugates might be largely determined by the immunogenicity of the 29 amino acids, irrespective of which region of the carrier protein is attached to the polysaccharide antigen (30), we determined the minimum and optimal motif recognized by PglL.

**FIG 4:**
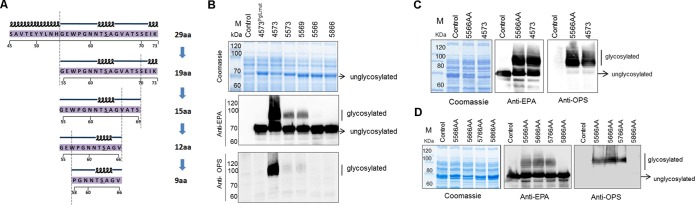
Determination of the minimum recognition motif for the O-linked glycosylation system. (A) A schematic of how the sequence of PilE 45S-73K was shortened step by step from 29 amino acids (aa) down to 9 amino acids, according to its secondary structure. (B) Coomassie blue staining and Western blotting assays were used to analyze the glycosylation of the sequences depicted in panel A. 4573 indicates a fusion of the S^45^-K^73^ fragment at the N terminus of rEPA (pET-pglL-rEPA^4573N^), and other fusion patterns are indicated similarly. The control was strain 301DWP lacking plasmid, and 4573^PglLmut^ indicates the expression of PglL_mut_ (pET-pglL_mut_-rEPA^4573N^). (C) Two hydrophilic fragments were added before and after the 12-amino-acid sequence (pET-pglL-rEPA^5566AA^), and glycosylation was assessed as in panel B. (D) The recognition motif was shortened to 10 amino acids (^57^WPGNNTSAGV^66^), based on 5566AA, and glycosylation was assessed as in panel B.

First, we truncated the sequence step by step according to its secondary structure (38) using the N-terminus-fused recombinant protein rEPA^4573N^ as a model (Fig. 4A). By using nested PCR (see Fig. S3 in the supplemental material), G55 to K73 (5573), G55 to S69 (5569), G55 to V66 (5566), and P58 to V66 (5866) were amplified, and new plasmids containing a single one of each of these sequences were created by replacing the fragment S45 to K73 in pET-PglL-rEPA^4573N^ with these sequences. These various expression vectors were then introduced into strain 301DWP. Results from Western blot analyses of these strains reveal that the amount of glycosylated protein decreased as the length of the peptide decreased. Hardly any glycosylated peptides were detected when the peptide was only 12 amino acids long (rEPA^5566^) (Fig. 4B). One possible reason for this might be that the loss of flanking regions inhibited the correct folding, and hence presentation, of the core recognition motif. Thus, we chose two hydrophilic fragments from the rEPA sequence (^592^DPRNVGGDLD^601^ and ^621^QPGKPPR^627^) to add before and after the 12-amino-acid sequence described above. As expected, glycosylation of this recombinant protein (named rEPA^5566AA^) was significantly improved compared with that of rEPA^5566^, and its level of glycosylation was comparable to that of rEPA^4573N^ (Fig. 4C). Based on this finding, we further shortened the recognition motif to ensure that the minimum recognition motif was obtained. By further deleting the amino acids one by one from both sides based on the rEPA^5566AA^ sequence (Fig. 4D; see also Fig. S4 in the supplemental material), we confirmed that a minimum recognition motif of 10 amino acids (^57^WPGNNTSAGV^66^) is required for efficient glycosylation.

### Optimization of the recognition motif of the O-linked glycosylation system.

**FIG 5:**
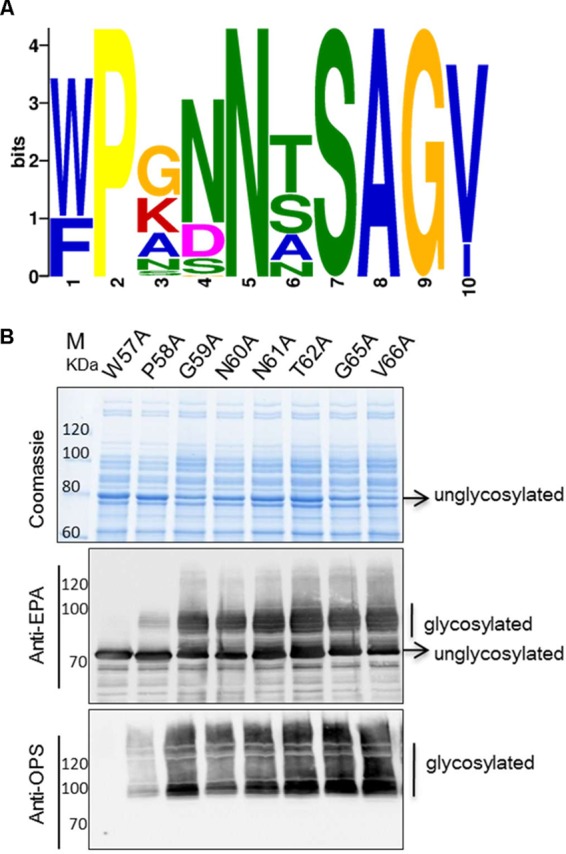
Analysis of the minimum recognition motif. (A) Conservation analysis of the 63 nonredundant PilE homolog sequences around S^63^ in *Neisseria meningitidis* (http://meme-suite.org/)*.* (B) Alanine scanning of the glycosylated sequence. Plasmids containing these sequences were transformed into strain 301DWP, and glycosylation was detected by Western blotting.

Next, we aimed to optimize the shortened sequence by shuffling some amino acids within the minimum core motif. First, in the NCBI protein database, a total of 63 nonredundant homolog sequences of the native carrier protein PilE of *N. meningitidis* were aligned for a conservation analysis of the shortened motif. According to the results, the seventh amino acid, S, was the site of glycosylation, and the second, fifth, eighth, and ninth amino acids supported the uniqueness of this motif (Fig. 5A). Therefore, we performed alanine scanning to rapidly identify the importance of particular residues for glycosylation. All amino acids from W57 to V66, except S63 and A64, were mutated to alanine one by one, and Western blot analyses were used to detect glycosylated proteins.

To our surprise, the glycosylation of rEPA was abolished only when W57 was mutated to alanine, even though decreased glycosylation levels were evident when P58 was mutated to alanine instead of when the amino acids nearer S63 were mutated (Fig. 5B). That is, several of the other amino acid residues had no significant influence on glycosylation. In accordance with the results of the previous conservation analysis, W57 was not unique, and in some cases, it was replaced with phenylalanine. To determine if the sequence containing phenylalanine could also be glycosylated, we generated a W57F variant. Although the difference between tryptophan and phenylalanine is only an aromatic ring, decreased levels of glycosylation were observed in the W57F variant (see Fig. S5 in the supplemental material).

**FIG 6:**
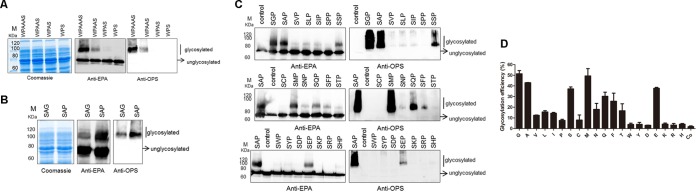
Optimization of the recognition motif. (A) The middle 4 amino acids (GNNT) of the recognition motif were replaced with single, double, or triple alanines or were removed completely. Plasmids containing these sequences were transformed into strain 301DWP, and glycosylation was detected by Western blotting. (B) A G65P mutant was constructed based on the sequence ^57^WPAAASAG^65^. This sequence was used and assessed as described for panel A. (C) The A after S^63^ was mutated into one of each of the other 19 amino acids. After plasmids with each of these sequences were transformed into strain 301DWP and protein expression was induced, periplasmic fractions were extracted and a Western blot analysis was performed to detect which sequences resulted in glycosylation. Control indicates the control group that expressed the carrier protein only (pET-pglL_mut_-rEPA^MOOR-AA^). (D) Western blotting assays were performed as in panel C. The glycosylated and unglycosylated bands of each lane were framed and used in a volume analysis performed by using the software Quantity One. The bar graph shows the percentage of glycosylated proteins for each transformant of 301DWP. “Co” indicates the control group, and the error bars indicate the standard deviations.

We had previously shown that each residue between ^57^WP^58^ and S^63^ (GNNT) had no obvious influence on glycosylation, implying that these residues may be interchangeable or dispensable. However, when we created a series of variants in which G, GN, N, or NN at 57 to 66 were deleted or the NN was mutated into an A, glycoproteins were hardly detectable (see Fig. S6A and B in the supplemental material), except in the cases with the amino acid sequence GAT (see also Fig. S6B in the supplemental material). Therefore, we performed further optimization, and the results from a Western blot analysis show that the peptide containing AAA between ^57^WP^58^ and S^63^ displayed a glycosylated serine (see Fig. S6C in the supplemental material). Finally, based on the WPAAAS sequence, we generated sequences with all combinations of A residues (no A, a single A, a double A [AA], or a triple A [AAA]) between ^57^WP^58^ and S^63^. As the number of alanine residues decreased, so did the level of protein glycosylation (Fig. 6A). From this set of experiments, we concluded that the optimal amino acid sequence between ^57^WP^58^ and S^63^ was AAA.

Our previous work confirmed that the number of amino acids after S^63^ influences glycosylation (see also Fig. S4 in the supplemental material). We achieved the same result here when the amino acids between ^57^WP^58^ and S^63^ were mutated to AAA (see Fig. S7A in the supplemental material). Therefore, we suspected that the amino acids after S form another flexible structure to separate the recognition motif from the downstream domains. In some studies in eukaryotes, proline residues have had a positive effect on O-linked glycosylation. Thus, we hypothesized that if we added an inflexible proline, it would inhibit this type of unexpected secondary structure. To test this idea, we created a variant based on the WPAAASAG sequence by replacing G^65^ with a proline. The results of a Western blot analysis indicate that this change induced increased levels of glycoproteins (Fig. 6B). In contrast, further deleting A^64^ abolished all glycosylation (see Fig. S7B in the supplemental material).

Based on these findings, we hypothesized that a simple and efficient sequence for O-glycosylation would be W-P-Xn-S-Xm-P, in which Xn is a flexible amino acid and AAA is optimal. To test if the alanine between S^63^ and the last proline is optimal for glycosylation, we evaluated the glycosylation efficiencies following the mutation of A^64^ to each of the other 19 amino acids in turn. After induction, periplasmic fractions were extracted and Western blot analyses were performed to detect glycoprotein. The results show that a higher glycosylation efficiency could be achieved only when glycine, alanine, or methionine was inserted between S^63^ and the last proline (Fig. 6C). Additionally, almost no glycoprotein was detectable when proline, cysteine, aspartic acid, or lysine was inserted (Fig. 6C). To evaluate the glycosylation efficiency, the bands of glycosylated and unglycosylated proteins were quantized with gray-scale values by using the software Quantity One. These results show that higher glycosylation efficiency (>40%) could be achieved only when glycine, alanine, or methionine was inserted between S^63^ and the last proline (Fig. 6D). Furthermore, an ELISA (see also Text S1 in the supplemental material), which used the extracted periplasmic fractions to coat 96-well plates and detected the optical density at 490 nm (OD_490_) of EPA and OPS using anti-EPA and anti-OPS antibodies, was also performed, and its results were used to calculate the glycosylation effect relative to the control group; these findings were similar to the Western blot assay results (see Fig. S7C in the supplemental material). Taking into account the stability of the structure, the final recognition motif was optimized to be 8 amino acids long (WPAAASAP) and was named the MOOR (minimum optimal O-linked recognition) motif.

When conjugate vaccines are produced by chemical methods, the substrate proteins are usually toxin proteins and the glycans can combine on many protein surface regions with strong immunogenicity. The acquisition of a minimum motif makes the concatenation of MHC binding peptides possible, and we believe that this may be an important step forward in the design of bioconjugates. We attempted to replace the hydrophilic fragments around the MOOR with different peptides that are recognized by MHC-II, such as gp100^44–59^ (WNRQLYPEWTEAQRLD) (39), HA^307–319^ (PKYVKQNTLKLAT) (40), P30 (TT^947–967^; FNNFTVSFWLRVPKVSASHLE) (41, 42), and P2 (TT^830–843^; QYIKANSKFIGITE) (41, 42), to design the glycan peptide. For example, the hydrophilic fragment at the C terminus of the core glycosylation sequence was replaced with gp100^44–59^, HA^307–319^, or P30, and the hydrophilic fragments at the N and C termini of the core glycosylation sequence were replaced with P30 and P2, respectively. We then tested the glycosylation of these designed carrier proteins, and the results of Western blot analyses show that all combinations were efficiently glycosylated (see Fig. S8 in the supplemental material). Evaluation of the immune response induced by these recombinant bioconjugates is under way in our laboratory.

## DISCUSSION

We demonstrated that O-linked glycosylation system were a better way to create conjugate vaccines, and we further optimized the glycosylation recognition motif for this system. We found that CTB is a better carrier protein than rEPA and that glycosylated CTB, similar to natural CTB, is a polymer. Because polymeric particles may lead to a preferred interaction with antigen-presenting cells (43), glycosylated CTB has the potential to be an ideal carrier protein. An example of using polymeric particles to create vaccine was hepatitis B virus core particle, which, acting as a carrier, was fused with a conserved region of human influenza A virus to create a vaccine; it provided complete protection against lethal challenge in animal experiments (44–46). Potentially, particle carriers could also be applied to conjugate vaccines in the future.

Glycoproteins are thymus-dependent antigens which can induce T-cell, Ig isotype switch, and memory responses against the polysaccharide moiety. In this process, complement-activating, bactericidal IgG1 can be produced. In our animal experiments, most of the IgG antibody produced was subtype IgG1. Similarly, a previous clinical study of a pneumococcal conjugate vaccine also showed that IgG1 was the main antibody subtype elicited in infants following vaccination (47). So, conjugate vaccines show a better immune reaction than previous polysaccharide vaccines which are thymus-independent antigens and the main inducers of IgM production.

Although the production of bioconjugate vaccines is still in its initial stages, it offers huge advantages over chemical methods, as previously discussed (16, 48, 49). Currently, there are two known types of glycosyltransferases (PglL and PglB), both with the potential to produce conjugate vaccines. PglB has been more widely employed to date, mainly owing to the fact that its glycosylation recognition sequence, a 5-amino-acid motif, has been clearly defined. However, some polysaccharides are not suitable for PglB. Group I and IV capsules and OPS can synthesize UndPP (50), but some, including 50 of 54 known structural capsules in *S. pneumoniae* (20), 24 of 35 structural capsules in *S. suis* (21, 22), and 9 of 47 OPSs in *Salmonella* (23, 36), also lack appropriate reducing ends and cannot be transferred in N-linked glycosylation systems. Recently, a study showed that by the mutation of some amino acids in PglB, a *Salmonella* Gal-initiated O antigen could be transferred to proteins (51). Compared with PglB, PglL has more potential applications because of its lower structural specificity for sugar substrates and because it can transfer virtually any glycan *in vivo* (26). For example, a heptavalent conjugate vaccine, Prevnar, which is produced by chemical methods and consists of conjugates of diphtheria toxin mutant (CRM197) and 4, 6B, 9V, 14, 18C, 19F, and 23F capsular polysaccharides, is a successful vaccine for pneumococcal disease and has been licensed in more than 100 countries around the world. The results of clinical trials prove that this heptavalent pneumococcal conjugate vaccine successfully prevents pneumococcal infections in children (52). Theoretically, our O-glycosylation system also could be used to create *S. pneumoniae* conjugate vaccines because the capsular polysaccharides are similarly synthesized through the Wzy-dependent pathway (20). Of course, many difficulties, such as protein expression, remain to be addressed.

Here, we also elucidated that an 8-amino-acid motif named MOOR (sequence WPAAASAP) is sufficient for glycosylation. Statistical analyses of the different peptides containing O-glycans show that proline residues in positions −3 and +1 of the glycosylation sites have a positive effect on eukaryotic glycosylation (53). However, we found that proline at positions −4 and +2 increased glycosylation in prokaryotes. This motif is similar to a sequence in protein AniA (28), whose O-glycosylation sites are the S residues in the sequence ^367^GAAPAASAPAASAP^380^. Given that this sequence differs significantly from ^45^WPGNNTSAGV^73^, we propose that one reason for the diversity in natural O-glycosylation is that the amino acids surrounding serine may have other biological functions, such as delivering an external signal. Our motif provides new insight into the use of O-linked glycosylation for the biological production of conjugate vaccines.

The biological strategy for producing conjugate vaccines directly in attenuated strains described here is different from previously reported biological methods (16), in which three vectors need to be constructed and introduced into *E. coli*. In contrast, only one vector is needed in our strategy, and so the process is easier and more convenient. Not only could the production of conjugate vaccines directly in attenuated strains avoid the difficulty of cloning a long polysaccharide gene cluster, but this strategy could also be applied in strains whose polysaccharide gene cluster remains unknown. In summary, using O-linked glycosylation to produce polysaccharide conjugate vaccines in attenuated strains will be a breakthrough in bacterial vaccine production.

## MATERIALS AND METHODS

### Animals.

All animals were purchased from the Laboratory Animal Center of the Academy of Military Medical Sciences and were also housed in the Center, which had a constant ambient temperature (23 ± 3°C) and humidity (55% ± 5%). Food, bedding, and water were changed every 4 days. All animal experiments were approved by and performed in accordance with the recommendations of the Academy of Military Medical Sciences Institutional Animal Care and Use Committee.

### Strains, growth conditions, and plasmids.

All bacterial strains were grown in lysogeny broth medium or on solid medium containing 1.5% agar. *Escherichia coli* DH5α was used in cloning experiments. Cells were cultured at 37°C to an OD_600_ of 0.4 to 0.5, and 1 mM IPTG was used at 30°C for 10 h to induce protein expression. The main plasmids used in this study are listed in Table 1. PglL_mut_ was constructed using a Fast Mutagenesis System kit (Transgen Biotech) according to the manufacturer’s instructions, with primers pglL-C-A-F/pglL-C-A-R (see Table S1 in the supplemental material) to mutate the 57th base, C, to an A. This resulted in a TAA sequence, which could stop protein translation. The vectors used in the glycosylation site optimization experiments were created based on pET-pglL-rEPA^4573N^ using nested PCR (see also Fig. S3 in the supplemental material).

### LPS silver staining.

*Shigella flexneri* 2a strains, cultured at 37°C in 5 ml of lysogeny broth medium for 12 h, were washed with phosphate-buffered saline (PBS) three times and then resuspended in 100 µl of buffer (2% [wt/vol] sodium dodecyl sulfate [SDS], 4% [vol/vol] 2-mercaptoethanol, 1 M Tris-HCl, pH 6.8, and 0.02% [wt/vol] bromophenol blue). After boiling in a water bath for 10 min, 4 mg of proteinase K was added and incubated at 60°C for 2 h. The whole-cell lysates were then separated by SDS-PAGE. LPS was fixed in the gel with 40% (vol/vol) ethanol and 10% (vol/vol) acetic acid for 30 min and then sensitized for 30 min in a solution containing 7% (wt/vol) sodium acetate, 0.2% (wt/vol) sodium thiosulfate, 30% (vol/vol) ethanol, and 0.25% (vol/vol) glutaraldehyde. The gels were subsequently washed with double-distilled water (ddH_2_O) three times for 10 min and were then stained with 100 ml of staining solution (0.25 g of silver nitrate and 40 µl of formaldehyde) for 20 min. The gels were washed twice for 1 min each and then transferred into 100 ml of solution (2.5 g of sodium carbonate, 3 µl of 5% [wt/vol] sodium thiosulfate, and 40 µl of formaldehyde). The reactions were stopped with 1.5% (wt/vol) EDTA-Na_2_ for 10 min followed by repeated washings with ddH_2_O. All solutions were prepared with ddH_2_O.

### Western blot analyses.

After protein expression was induced with IPTG, whole-cell extracts were heated to 100°C with 1× SDS-PAGE loading buffer containing 50 mM Tris-HCl (pH 6.8), 1.6% (wt/vol) SDS, 0.02% (wt/vol) bromophenol blue, 8% (vol/vol) glycerol, and 20 mM dl-dithiothreitol. Proteins were separated by SDS-PAGE, and Western blot analysis was performed using polyvinylidene fluoride membranes (GE Healthcare) as previously described (54). Anti-6×His antibodies conjugated to horseradish peroxidase (HRP) (Abmart) were used to detect the proteins that contained a 6×His tag. Anti-EPA antibody (P2318; Sigma) (1:7,500) was used to detect rEPA and rEPA mutants. Antiserum (catalog no. 210227; Denka Seiken) (1:50) specific for the *S. flexneri* 2a OPS was used to detect the glycans of glycoproteins. Both the anti-EPA antibody and the antiserum were produced in rabbits, and HRP-conjugated anti-rabbit IgG (Transgen Biotech) was used as the secondary antibody.

### **Production and purification of glycosylated proteins**.

The IPTG-induced bacteria were harvested by centrifugation (at 7,000 × *g* for 10 min), and the resulting cell pellets (10 g) were resuspended in buffer A1 (20 mM Tris-HCl, pH 7.5, 10 mM imidazole, and 500 mM NaCl) to a volume of 100 ml, after which the cells were broken by sonication. Following sonication, the product was centrifuged at 12,000 × *g* for 10 min, and the resulting supernatant, which contained CTB-OPS, was applied on a chelating column (φ, 1.6 by 15 cm; GE Healthcare) that had been equilibrated with buffer A1. After the column was washed with buffer A1, the CTB-OPS sample was eluted with 100% buffer B1 (20 mM Tris-HCl, pH 7.5, 500 mM imidazole, and 500 mM NaCl). The eluent was desalted through a G25 column (φ, 2.5 by 30 cm; GE Healthcare) with buffer A3 (20 mM HAc-NaAc, pH 5.4) or, for rEPA-OPS, with buffer A2 (20 mM Tris-HCl, pH 7.5) and then applied to a ProteinPak SP8HR column (φ, 1 by 10 cm; Waters) or, for rEPA-OPS, to a ProteinPak DEAE8HR column (φ, 1 by 10 cm; Waters). After the ProteinPak SP8HR (DEAE8HR for rEPA-OPS) column was washed with buffer A3, or with buffer A2 for rEPA-OPS, the glycoprotein sample was eluted with a gradient of 0 to 100% buffer B3 (20 mM HAc-NaAc, pH 5.4, and 1 M NaCl) or of buffer B2 (20 mM Tris-HCl, pH 7.5, and 1 M NaCl) for rEPA-OPS. The elutriated samples were analyzed by Western blotting assays, and the sample containing protein-OPS was separated by Superdex 75 fast-performance liquid chromatography (FPLC) (φ, 1 by 30 cm; GE Healthcare) with buffer A4 (20 mM phosphate buffer, pH 7.4, and 150 mM NaCl).

### Immunization experiments.

Groups of 10 female BALB/c mice, 5 weeks old, were used in immunization experiments. The purified bioconjugates were diluted with PBS and mixed with alum (10%; General Chemical). The dose of polysaccharide was 2.5 µg per injection. Immunizations were performed on days 1, 15, and 29. Blood samples were taken by tail snip on day 39, and the serum was stored at 4°C.

### ELISA.

Ninety-six-well immunoplates were coated overnight with 100 µl of 100 µg/ml LPS from *S. flexneri* 2a strain 301 diluted in carbonate coating buffer (50 mM Na_2_CO_3_-NaHCO_3_, pH 9.6) at 4°C. The wells were then washed with 200 µl of wash buffer (1× PBS with 0.05% Tween 20) three times, and the plates were patted dry. Then, 200 µl of ELISA blocking buffer (1× PBS with 5% milk) was added to each well, and the plates were incubated at 37°C for 2 h. After washing and drying, 100 µl of immune serum, diluted to different concentrations in dilution buffer (1× PBS with 0.5% milk), was added to each well and incubated for 1 h at 37°C. After another washing and drying step, 100 µl of HRP-conjugated goat anti-mouse IgG antibodies (Abcam) diluted 1:50,000 in dilution buffer was added to each well, and the plates were incubated for 1 h at 37°C. After each well was washed five times and dried, 100 µl of color solution (*o*-phenylenediamine [OPD]–H_2_O_2_ solution) was added, and the samples were left to react in the dark for 15 min at room temperature. The reaction was stopped with 50 µl of ELISA stop solution (2 mol/liter H_2_SO_4_). A microplate reader was used to measure the OD_490_.

### Complement sterilization experiments.

*S. flexneri* 2a strain 301 was cultured at 37°C to an OD_600_ of approximately 2.0 and then diluted 40,000-fold with normal saline. Immune sera for each group were mixed and incubated at 56°C for 30 min to inactivate complement and then diluted to various concentrations. After the addition of 10 µl immune serum (using normal saline as a negative control) to 10 µl diluted cells, the mixture was incubated for 1 h at room temperature, and then 20 µl of complement was added to each group. The samples were incubated at 37°C for 1 h, plated on LB solid medium, and cultured overnight at 37°C. The number of single colonies was counted to calculate the percentage of survivors.

### Survival experiment.

A survival experiment was performed on day 43 (14 days after the last injection). *S. flexneri* 2a strain 301 was cultured at 37°C at an OD_600_ of 2.0 and then diluted with normal saline to approximately 3 × 10^7^ CFU/200 µl. The immunized mice (described above) were infected intraperitoneally with 200 µl/mouse of the diluted strain and then observed for death every 8 h.

### Preparation of periplasmic extracts.

Periplasmic extracts were prepared by osmotic shock lysis as described previously (9). Briefly, 5 ml of IPTG-induced cells was harvested by centrifugation and washed with PBS twice. The cells were then incubated in 100 µl of buffer (30 mM Tris-HCl, pH 8.5, 20% [wt/vol] sucrose, 1 mM EDTA, and 1 mg/ml lysozyme) on ice for 30 min and centrifuged for 20 min to yield periplasmic proteins in the supernatants.

## SUPPLEMENTAL MATERIAL

Text S1  Supplemental methods. The supplemental methods used in our experiments included the construction of ligase-defective strains, plaque assays, Sereny tests, determination of sugar content, and calculation of the relative glycosylation. Download Text S1, PDF file, 0.2 MB

Figure S1  Toxicity test for strain 301DWP. (A) PCR was performed to amplify the virulence factors (IpaA, IpgB, MxiD, and VirG), located in the virulence plasmid from *S. flexneri* 2a strains 301 and 301DWP. Bands in the marker lane from top to bottom show fragments of 7,000, 5,000, 3,000, 2,000, 1,000, and 500 bp. (B) Sereny test in guinea pigs at 36 and 48 h postinfection. Representative images of the cornea after infection with *S. flexneri* 2a strain 301, 301ΔwaaL, or 301DWP. Control animals were treated with normal saline. (C) Plaque assays were performed to detect the virulence of *S. flexneri* 2a strains 301, 301ΔwaaL, and 301DWP in HeLa cells. The diameters of the plaques were measured using a microscope. Bar, 1,000 µm. Download Figure S1, PDF file, 0.1 MB

Figure S2 O-linked glycosylation in *E. coli* strain CLM24. Western blot analysis with anti-His antibodies to detect glycosylation in *E. coli* strain CLM24 coexpressing WbbL (pACU184-wbbL) and the recombinant substrate protein rEPA^4573N^ (pMM-rEPA^4573N^), with or without glycosyltransferase PglL (pET-PglL). Download Figure S2, PDF file, 0.1 MB

Figure S3 Using nested PCR to mutate the amino acids in the glycosylation sequence. The upper line is a schematic of pET-pglL-rEPA^4573N^, which contains a SacI site at the C terminus of *Ptac* in rEPA and a SpeI site between 4573 and rEPA. We designed an upstream primer containing a SacI site and downstream primers 1, 2, and 3 containing a SpeI site, as shown in the bottom line. pET-pglL-rEPA^4573N^ was amplified using nested PCR to mutate position 4573. Download Figure S3, PDF file, 0.04 MB

Figure S4 Glycosylation of the sequences truncated after S^63^. After shortening of the original glycosylation sequence to 10 amino acids, the amino acids from the C terminus were deleted one by one, which left three (AGV), two (AG), or one (A) amino acid after S^63^. Plasmids containing the mutant sequences were transformed into strain 301DWP, and the amounts of glycosylated protein in each group were assessed by Western blotting. Download Figure S4, PDF file, 0.2 MB

Figure S5 Glycosylation status when W^57^ was replaced with F. W^57^ in the ^57^WPGNNTSAGV^66^ sequence (pET-pglL-rEPA^5766AA^) was mutated to F, and either the original sequence or the W57F mutant sequence was transformed into strain 301DWP. Western blot analyses with anti-EPA (middle) and anti-OPS (right) antibodies were performed to compare the glycosylation statuses between transformed 301DWP strains. The corresponding gel stained with Coomassie blue is shown on the left. Download Figure S5, PDF file, 0.2 MB

Figure S6 Optimization of the amino acids between ^57^WP^58^ and S^63^. New plasmids were generated by deleting specific residues, and then these plasmids were transformed into strain 301DWP, and glycosylation was detected by Western blot analysis. (A) The G or GN of the original sequence GNNT (control) between ^57^WP^58^ and S^63^ was deleted, resulting in NNT or NT. (B) One or two of the N residues between ^57^WP^58^ and S^63^ were deleted, or the remaining N was mutated into an A, resulting in the amino acid sequence GNT, GT, or GAT. (C) Based on the results from GAT in panel B, the G alone or both the G and the T were mutated into A’s, and so the amino acids between ^57^WP^58^ and S^63^ became GAA or AAA. Download Figure S6, PDF file, 0.2 MB

Figure S7 Glycosylation status of sequences that were truncated or mutated after S^63^. (A) Based on the sequence WPAAASAGV, amino acids from the C terminus were deleted one by one, leaving three (AGV), two (AG), or one (A) amino acid after S^63^. Plasmids containing the mutant sequences were transformed into strain 301DWP, and the amounts of glycosylated proteins in each group were assessed by Western blotting. (B) Based on the sequence containing amino acids AAA between ^57^WP^58^ and S^63^, two new plasmids were created by mutating the original AG after S^63^ into AP or P. Plasmids containing the mutant sequences were transformed into strain 301DWP, and the amounts of glycosylated proteins in these two groups were assessed by Western blotting. (C) The A after S^63^ was mutated into one of each of the 19 other amino acids. After plasmids with each of these sequences were transformed into strain 301DWP and protein expression was induced, the extracted periplasmic fractions of the stains were used to coat 96-well plates. The OD_490_s of EPA and OPS were measured by ELISAs using anti-EPA and anti-OPS antibodies. “Co” indicates the control group. The glycosylation level in each group was compared with that of the control, and the histogram indicates the relative glycosylation levels. The dotted line indicates the corresponding value of the control group. Download Figure S7, PDF file, 0.1 MB

Figure S8 Glycosylation status of sequences with different peptides in the flanking scaffold sequence. (A) The hydrophilic fragment at the C terminus of the MOOR was replaced with HA^307–319^ (PKYVKQNTLKLAT). The mutant plasmid was transformed into strain 301DWP, and a Western blot analysis was used to detect glycosylation. The control expressed the carrier protein alone. (B) The hydrophilic fragment at the C terminus of the core glycosylation sequence was replaced with gp100^44–59^ (WNRQLYPEWTEAQRLD). This sequence was used and assessed as described in panel A. (C) The hydrophilic fragments at the N and C termini of the core glycosylation sequence were replaced with P30 (TT^947–967^; FNNFTVSFWLRVPKVSASHLE) and P2 (TT^830–843^; QYIKANSKFIGITE), respectively, or the C terminus only was replaced with P30. Glycosylation was detected as described in panels A and B. Download Figure S8, PDF file, 0.05 MB

Table S1  Primers used in our experiments. Sequences and the introductions about the primers used in our experiments. The underlined bases indicate restriction sites. Table S1, PDF file, 0.2 MB
