# Supplementary material for: Biosynthesis of Conjugate Vaccines Using an O-Linked Glycosylation System
Source: mBio. 2016 Apr 26;7(2):e00443-16. doi: 10.1128/mBio.00443-16 (PMC4850263; doi:10.1128/mBio.00443-16)
Supplement: Text S1 — Supplemental methods. The supplemental methods used in our experiments included the construction of ligase-defective strains, plaque assays, Sereny tests, determination of sugar content, and calculation of the relative glycosylation. Download [file mbo002162786s1.pdf]

Supplemental methods

Construction of ligase-defective strains. Deletion of the *waaL* chromosomal gene was performed as described by Datsenko and Wanner (Datsenko KA, Wanner BL. 2000. Proc Natl Acad Sci U S A 97:6640-6645.) with minor modifications. Using the NCBI database of *waaI* (*waaL*) gene sequences from *S. flexneri* 2a strain 301, two approximately 500-bp fragments adjacent to the gene targeted for deletion were obtained as a linear DNA fragment through amplification using upstream and downstream primers: waaIUP1/waaIUP2 and waaIDP1/waaIDP2 (Supplementary Table 1). These two fragments were inserted into pET-kan, which contains a kanamycin resistance gene flanked by FRT (FLP recognition target) sites, creating the plasmid pET-Up-kan-Down. The primers waaIUP1 and waaIDP2 were used to amplify the template DNA from pET-Up-kan-Down, and the products were introduced into *S. flexneri* 2a 301/pKD46 competent cells by electroporation. *E. coli* O157ΔwaaL and *Salmonella* paratyphi A strain CMCC 50973ΔwaaL were created using the same method.

Plaque assays. Plaque assays were performed as described previously (Oaks EV, Wingfield ME, Formal SB. 1985. Infect Immun 48:124-129.). HeLa cells were seeded onto 6-well plates in 2 ml of tissue culture medium per well at 37 ℃ in a humidified atmosphere of 5% CO_2:95% air. When the HeLa cells in each well were 80–90% confluent, Dulbecco’s modified Eagle’s medium without antibiotics or fetal bovine serum was used to wash the monolayers three times. The diluted bacterial suspension (2 ml) was added to the monolayer, and the cells were incubated at 37 ℃ for 90 min. During the incubation, the plates were rocked every 15 min. Next, the bacteria were aspirated and 2 ml of Dulbecco’s modified Eagle’s medium containing 5% fetal bovine

serum, 0.05% gentamicin, and 0.5% agarose was added to each well. After incubation at 37 ℃ in a humidified 5% CO_2:95% air atmosphere for 72 h, the agarose was removed and 1 ml of 1% (wt/vol) crystal violet was added to each well for 15 min. The diameters of the plaques were measured using a phase contrast microscope.

Sereny tests. The kerato-conjunctivity test (Sereny test) in guinea pigs was performed as reported previously (Kopecko DJ. 1994. Methods Enzymol 235:39-47.) (Hanson CE, Ruble GR, Essiet I, Hartman AB. 2001. Comp Med 51:224-229.). A total of 25 μl of *S. flexneri* 2a strain 301 (about 5.0×10^8 colony-forming units (CFU)) in phosphate-buffered saline (PBS) was micro-pipetted onto the conjunctiva of the eye, and the amount of inflammation was observed after 36 and 48 h.

Determination of sugar content. Sugar quantitation experiments were performed following an anthrone-sulfuric acid method. Briefly, 4 ml of anthrone-concentrated sulfuric acid (2 mg/ml^{-1}) was added to 1 ml of glucose solution of differing concentrations (0, 5, 10, 20, 30, 40, 60, or 80 $\mu g/ml^{-1}$), 50 $\mu l/ml^{-1}$ OPS of *S. flexneri* 2a strain 301, and samples. After 10 min in a boiling water bath, the absorbance at OD_{620} of the samples was measured on a microplate reader and Excel software was used to draw a standard curve line and calculate the sugar content.

Calculation of the relative glycosylation. To calculate the relative glycosylation, ELISA was used. Briefly, 96-well plates were coated with periplasmic fractions diluted in carbonate coating buffer. Total EPA and OPS were measured using an anti-EPA antibody (1:20,000) and anti-*S. flexneri* 2a OPS serum (1:40), respectively. HRP-conjugated goat anti-rabbit antibody (Transgen Biotech) diluted 1:5,000 in dilution buffer was used as the secondary antibody. Three

biological replicates were performed for each sample. A microplate reader was used to measure the absorbance at OD_{490}, and the resulting data were used in the formula ($\frac{\mathrm{OPS}/\mathrm{EPA(each\ group)}}{\mathrm{OPS}/\mathrm{EPA(control\ group)}}$), which showed relative glycosylation compared with that in the control group.
