# Supplementary figures and images for: Biosynthesis of Conjugate Vaccines Using an O-Linked Glycosylation System
Source: mBio. 2016 Apr 26;7(2):e00443-16. doi: 10.1128/mBio.00443-16 (PMC4850263; doi:10.1128/mBio.00443-16)

FIG S1

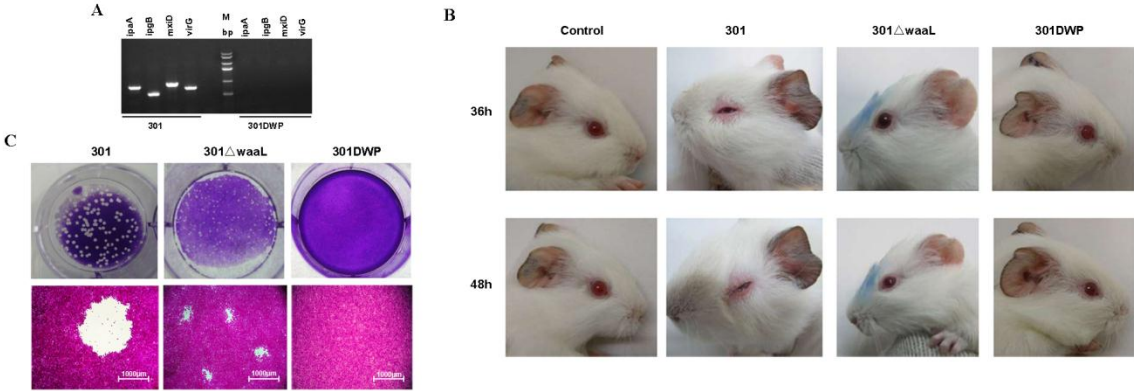

Supplement: Figure S1 — Toxicity test for strain 301DWP. (A) PCR was performed to amplify the virulence factors (IpaA, IpgB, MxiD, and VirG), located in the virulence plasmid from S. flexneri 2a strains 301 and 301DWP. Bands in the marker lane from top to bottom show fragments of 7,000, 5,000, 3,000, 2,000, 1,000, and 500 bp. (B) Sereny test in guinea pigs at 36 and 48 h postinfection. Representative images of the cornea after infection with S. flexneri 2a strain 301, 301ΔwaaL, or 301DWP. Control animals were treated with normal saline. (C) Plaque assays were performed to detect the virulence of S. flexneri 2a strains 301, 301ΔwaaL, and 301DWP in HeLa cells. The diameters of the plaques were measured using a microscope. Bar, 1,000 µm. Download [file mbo002162786sf1.pdf]

**FIG S2**

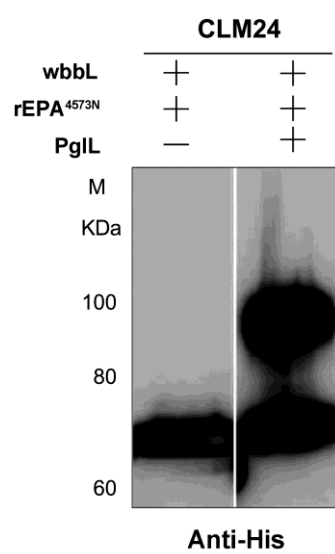

Supplement: Figure S2 — O-linked glycosylation in E. coli strain CLM24. Western blot analysis with anti-His antibodies to detect glycosylation in E. coli strain CLM24 coexpressing WbbL (pACU184-wbbL) and the recombinant substrate protein rEPA4573N (pMM-rEPA4573N), with or without glycosyltransferase PglL (pET-PglL). Download [file mbo002162786sf2.pdf]

**FIG S3**

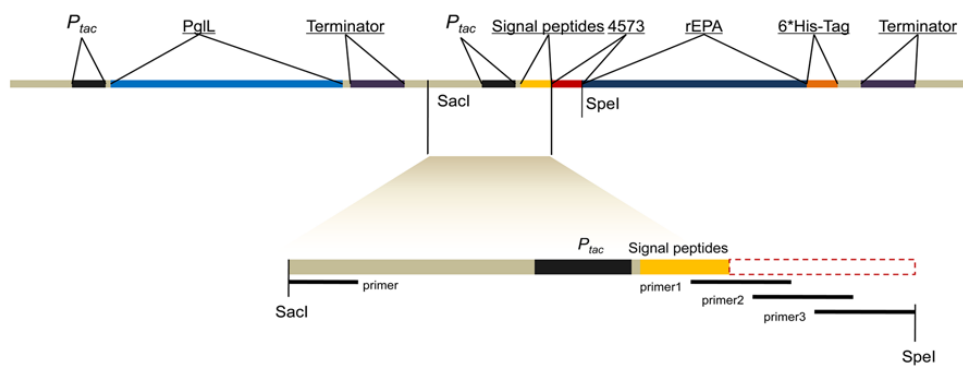

Supplement: Figure S3 — Using nested PCR to mutate the amino acids in the glycosylation sequence. The upper line is a schematic of pET-pglL-rEPA4573N, which contains a SacI site at the C terminus of Ptac in rEPA and a SpeI site between 4573 and rEPA. We designed an upstream primer containing a SacI site and downstream primers 1, 2, and 3 containing a SpeI site, as shown in the bottom line. pET-pglL-rEPA4573N was amplified using nested PCR to mutate position 4573. Download [file mbo002162786sf3.pdf]

**FIG S4**

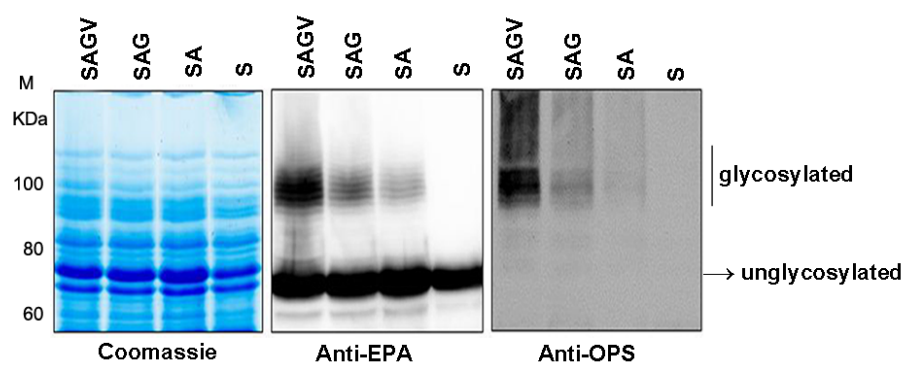

Supplement: Figure S4 — Glycosylation of the sequences truncated after S63. After shortening of the original glycosylation sequence to 10 amino acids, the amino acids from the C terminus were deleted one by one, which left three (AGV), two (AG), or one (A) amino acid after S63. Plasmids containing the mutant sequences were transformed into strain 301DWP, and the amounts of glycosylated protein in each group were assessed by Western blotting. Download [file mbo002162786sf4.pdf]

**FIG S5**

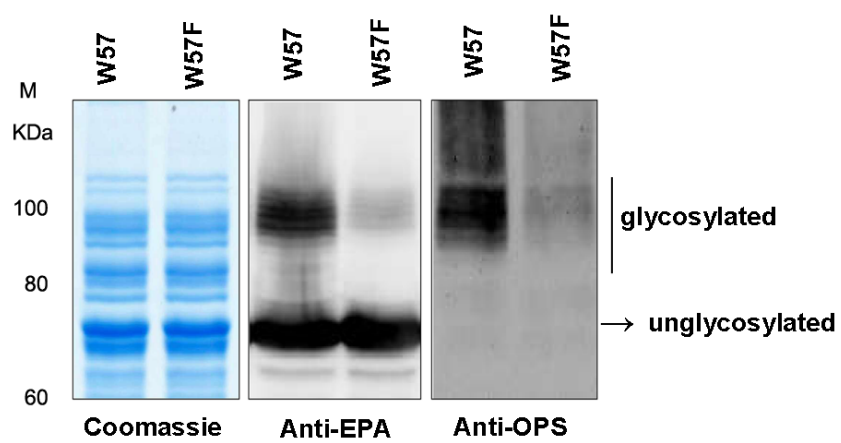

Supplement: Figure S5 — Glycosylation status when W57 was replaced with F. W57 in the 57WPGNNTSAGV66 sequence (pET-pglL-rEPA5766AA) was mutated to F, and either the original sequence or the W57F mutant sequence was transformed into strain 301DWP. Western blot analyses with anti-EPA (middle) and anti-OPS (right) antibodies were performed to compare the glycosylation statuses between transformed 301DWP strains. The corresponding gel stained with Coomassie blue is shown on the left. Download [file mbo002162786sf5.pdf]

**FIG S6**

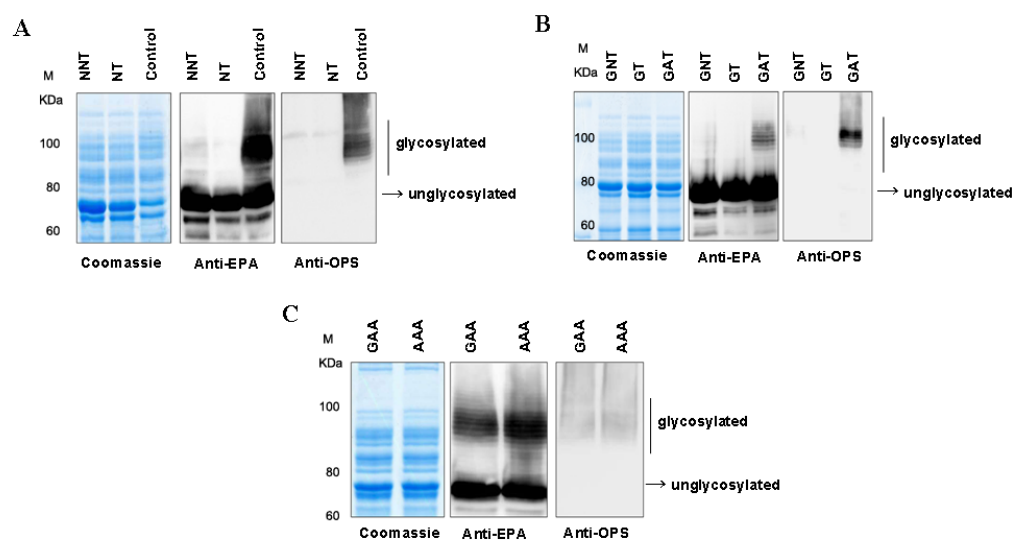

Supplement: Figure S6 — Optimization of the amino acids between 57WP58 and S63. New plasmids were generated by deleting specific residues, and then these plasmids were transformed into strain 301DWP, and glycosylation was detected by Western blot analysis. (A) The G or GN of the original sequence GNNT (control) between 57WP58 and S63 was deleted, resulting in NNT or NT. (B) One or two of the N residues between 57WP58 and S63 were deleted, or the remaining N was mutated into an A, resulting in the amino acid sequence GNT, GT, or GAT. (C) Based on the results from GAT in panel B, the G alone or both the G and the T were mutated into A’s, and so the amino acids between 57WP58 and S63 became GAA or AAA. Download [file mbo002162786sf6.pdf]

**FIG S7**

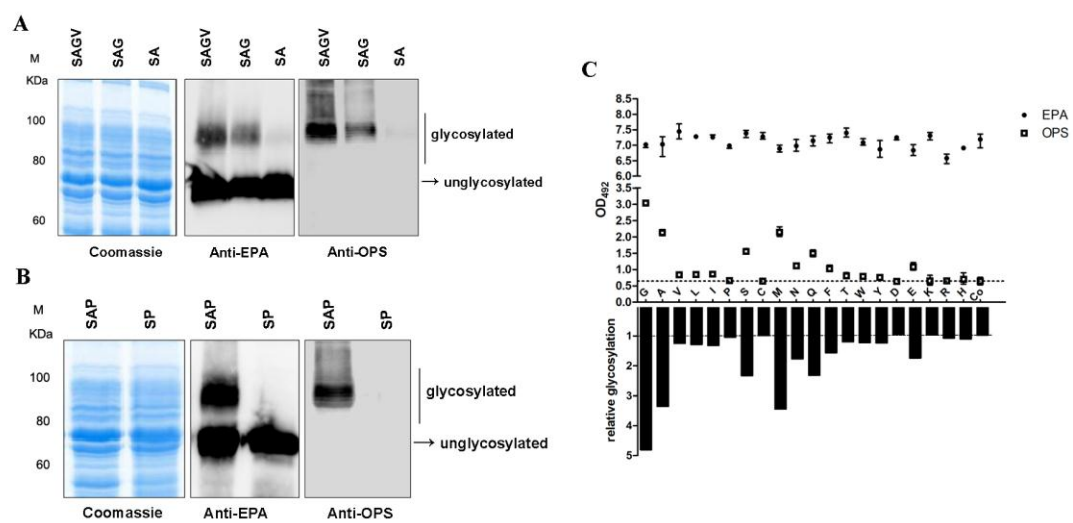

Supplement: Figure S7 — Glycosylation status of sequences that were truncated or mutated after S63. (A) Based on the sequence WPAAASAGV, amino acids from the C terminus were deleted one by one, leaving three (AGV), two (AG), or one (A) amino acid after S63. Plasmids containing the mutant sequences were transformed into strain 301DWP, and the amounts of glycosylated proteins in each group were assessed by Western blotting. (B) Based on the sequence containing amino acids AAA between 57WP58 and S63, two new plasmids were created by mutating the original AG after S63 into AP or P. Plasmids containing the mutant sequences were transformed into strain 301DWP, and the amounts of glycosylated proteins in these two groups were assessed by Western blotting. (C) The A after S63 was mutated into one of each of the 19 other amino acids. After plasmids with each of these sequences were transformed into strain 301DWP and protein expression was induced, the extracted periplasmic fractions of the stains were used to coat 96-well plates. The OD490s of EPA and OPS were measured by ELISAs using anti-EPA and anti-OPS antibodies. “Co” indicates the control group. The glycosylation level in each group was compared with that of the control, and the histogram indicates the relative glycosylation levels. The dotted line indicates the corresponding value of the control group. Download [file mbo002162786sf7.pdf]

**FIG S8**

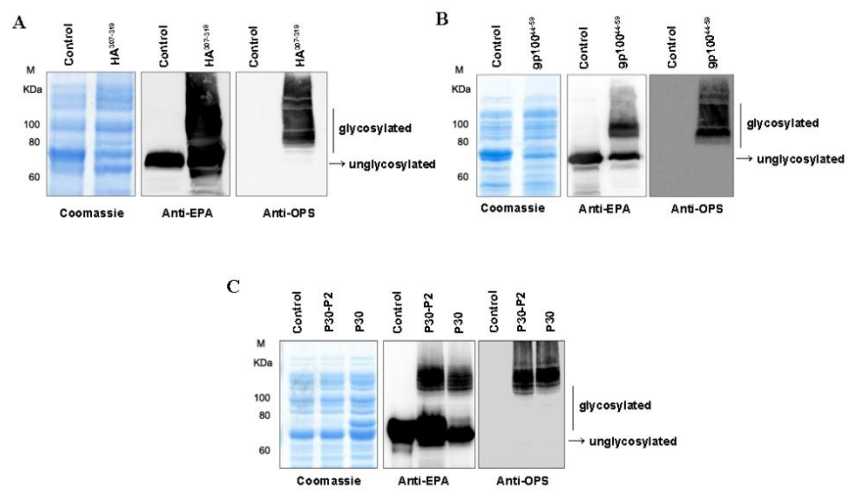

Supplement: Figure S8 — Glycosylation status of sequences with different peptides in the flanking scaffold sequence. (A) The hydrophilic fragment at the C terminus of the MOOR was replaced with HA307–319 (PKYVKQNTLKLAT). The mutant plasmid was transformed into strain 301DWP, and a Western blot analysis was used to detect glycosylation. The control expressed the carrier protein alone. (B) The hydrophilic fragment at the C terminus of the core glycosylation sequence was replaced with gp10044–59 (WNRQLYPEWTEAQRLD). This sequence was used and assessed as described in panel A. (C) The hydrophilic fragments at the N and C termini of the core glycosylation sequence were replaced with P30 (TT947–967; FNNFTVSFWLRVPKVSASHLE) and P2 (TT830–843; QYIKANSKFIGITE), respectively, or the C terminus only was replaced with P30. Glycosylation was detected as described in panels A and B. Download [file mbo002162786sf8.pdf]
