# Supplementary material for: Biosynthesis of Conjugate Vaccines Using an O-Linked Glycosylation System
Source: mBio. 2016 Apr 26;7(2):e00443-16. doi: 10.1128/mBio.00443-16 (PMC4850263; doi:10.1128/mBio.00443-16)
Supplement: Table S1 — Primers used in our experiments. Sequences and the introductions about the primers used in our experiments. The underlined bases indicate restriction sites. [file mbo002162786st1.pdf]

Table S1 Primers used in our experiments

Primers	Primer sequences(5′→3′)	Introduction
301waaIUP1	cgggatccgggtatgggaagaatcaag	Construction of ligase-defective strain
301waaIUP2	gcgtcgactcagaaatgctacggtgt	
301waaIDP1	ccaagctttggacctttagacaatcaa	
301waaIDP2	ccctcgagatgttaggaagcataccg	
50973waaIUP1	cgggatccaggctttgactatgtgga	Construction of ligase-defective strain
50973waaIUP2	gcgtcgacatctggcgatatgagtatg	
50973waaIDP1	ccaagctttagtgcaggcatattggg	
50973waaIDP2	ccctcgagtatcacctcgcagaacct	
PilE-U	cggaattcatgaacacccttcaaaaaggttt	Amplifiing PilE
PilE-D	cccaagcttttagtggtggtggtggtggtgtcc	
pglL-C-A-F	gccgccaaactgccgatttaaatcctgccctgcttc	Construction of $PglL_{mut}$
pglL-C-A-R	ttaaatcggcagtttggcggcggggtgcgcgccgga	
SacI-U	cgagctctgcataattcgtgtcgctcaaggcgcactc	
PstI-D	aactgcagggcagtttatggcgggcg	
**Primers below were applied to create mutation of amino acids by using nested-PCR SacI-U was the upstream primer for the following amplifications.**		
5573-D1(5569-D1)	agaagtgttgttgccgggccattcgccctcggccgccgatgcgctaaacgct	Shorten the sequence of glycosylation site
5573-D2	ggactagttttgatttcagaggaggttgccacgccggcagaagtgttgttgccggg	
5569-D2	ggactagtggaggttgccacgccggcagaagtgttgttgccggg	
5566-D	ggactagtcacgccggcagaagtgttgttgccgggccattcgccctcggccgccgatgcgctaaacgct	
5866-D	ggactagtcacgccggcagaagtgttgttgccgggctcggccgccgatgcgctaaac	
5566AA(5666AA,5766AA and 5866AA)-D1	gtcgaggtcgccgccgacgttgcgcgggtcctcggccgccgatgcgctaaacgct	
5566AA-D2	cacgccggcagaagtgttgttgccgggccattcgccgtcgaggtcgccgccga	
5666AA-D2	cacgccggcagaagtgttgttgccgggccattcgtcgaggtcgccgccgacgttg	
5766AA-D2	cacgccggcagaagtgttgttgccgggccagtcgaggtcgccgccgacgttg	
5866AA-D2	cacgccggcagaagtgttgttgccggggtcgaggtcgccgccgacgttg	
5566AA(5666AA,5766AA and 5866AA)-D3	ggactagtgcgcggcggtttgccgggctgcacgccggcagaagtgtt	
5566AA-D1	As shown above	Alanine scanning

5766AA-57A-D2	cgggctgcacgccggcagaagtgttgttgccgggcgcgtcgaggtcgccgccgacgt	of shortened glycosylation site
5766AA-58A-D2	cgggctgcacgccggcagaagtgttgttgccggcccagtcgaggtcgccgccgacgt	
5766AA-59A-D2	cgggctgcacgccggcagaagtgttgttggcgggccagtcgaggtcgccgccgacgt	
5766AA-60A-D2	cgggctgcacgccggcagaagtgttggcgccgggccagtcgaggtcgccgccgacgt	
5766AA-61A-D2	cgggctgcacgccggcagaagtggcgttgccgggccagtcgaggtcgccgccgacgt	
5766AA-62A-D2	cgggctgcacgccggcagaagcgttgttgccgggccagtcgaggtcgccgccgacgt	
5766AA-57A(58^62A)-D3	ggactagtgcgcggcggtttgccgggctgcacgccggcaga	
5766AA-65A,66A-D2	agaagtgttgttgccgggccagtcgaggtcgccgccgacgttg	
5766AA-65A-D3	ggactagtgcgcggcggtttgccgggctgcacggcggcagaagtgttgttgccgggcca	
5766AA-66A-D3	ggactagtgcgcggcggtttgccgggctgcgcgccggcagaagtgttgttgccgggcca	
5566AA-D1	As shown above	Optimization of the recognition motif between WP and S
NNT-D2	ccgggctgcacgccggcagaagtgttgttgggccagtcgaggtcgccgccgacgt	
NT-D2	ccgggctgcacgccggcagaagtgttgggccagtcgaggtcgccgccgacgt	
GNT-D2	ccgggctgcacgccggcagaagtgttgccgggccagtcgaggtcgccgccgacgt	
GT-D2	ccgggctgcacgccggcagaagtgccgggccagtcgaggtcgccgccgacgt	
GAT-D2	ccgggctgcacgccggcagaagtggcgccgggccagtcgaggtcgccgccgacgt	
GAA-D2	ccgggctgcacgccggcagaagcggcgccgggccagtcgaggtcgccgccgacgt	
WPAAAS(AAA)-D2	ccgggctgcacgccggcagaagcggcggcgggccagtcgaggtcgccgccgacgt	
WPAAS-D2	ccgggctgcacgccggcagaagcggcgggccagtcgaggtcgccgccgacgt	
WPAS-D2	ccgggctgcacgccggcagaagcgggccagtcgaggtcgccgccgacgt	
WPS-D2	ccgggctgcacgccggcagagggccagtcgaggtcgccgccgacgt	
NNT(NT, GNT, GT, GAT,GAA,	ggactagtgcgcggcggtttgccgggctgcacgccggcaga	

AAA, WPAAS, WPAS and WPS)-D3		
5566AA-D1	As shown above	Optimization of the recognition motif after S
SAG(SA, S, SAP and SP)-D2	agaagcggcggcgggccagtcgaggtcgccgccgacgt	
SAG-D3	ggactagtgcgcggcggtttgccgggctggccggcagaagcggcggcgggcca	
SA-D3	ggactagtgcgcggcggtttgccgggctgggcagaagcggcggcgggcca	
S-D3	ggactagtgcgcggcggtttgccgggctgagaagcggcggcgggcca	
SAP-D3	ggactagtgcgcggcggtttgccgggctggggggcagaagcggcggcgggcca	
SP-D3	ggactagtgcgcggcggtttgccgggctggggagaagcggcggcgggcca	
5566AA-D1	As shown above	Optimization of the first amino acid after S
SAG-D2	As shown above	
3A-AP-D3	ggactagtgcgcggcggtttgccgggctggggggcagaagcggcggcgggccagtcg	
3A-QP-D3	ggactagtgcgcggcggtttgccgggctggggttgagaagcggcggcgggccagtcg	
3A-FP-D3	ggactagtgcgcggcggtttgccgggctggggaaaagaagcggcggcgggccagtcg	
3A-DP-D3	ggactagtgcgcggcggtttgccgggctggggatcagaagcggcggcgggccagtcg	
3A-HP-D3	ggactagtgcgcggcggtttgccgggctggggatgagaagcggcggcgggccagtcg	
3A-KP-D3	ggactagtgcgcggcggtttgccgggctggggcttagaagcggcggcgggccagtcg	
3A-SP-D3	ggactagtgcgcggcggtttgccgggctggggactagaagcggcggcgggccagtcg	
3A-PP-D3	ggactagtgcgcggcggtttgccgggctggggtggagaagcggcggcgggccagtcg	
3A-WP-D3	ggactagtgcgcggcggtttgccgggctggggccaagaagcggcggcgggccagtcg	
3A-YP-D3	ggactagtgcgcggcggtttgccgggctggggataagaagcggcggcgggccagtcg	
3A-EP-D3	ggactagtgcgcggcggtttgccgggctggggttcagaagcggcggcgggccagtcg	
3A-RP-D3	ggactagtgcgcggcggtttgccgggctggggacgagaagcggcggcgggccagtcg	

3A-GP-D3	ggactagtgcgcggcggtttgccgggctggggtccagaagcggcggcgggccagtcg	
3A-VP-D3	ggactagtgcgcggcggtttgccgggctgggggacagaagcggcggcgggccagtcg	
3A-LP-D3	ggactagtgcgcggcggtttgccgggctgggggagagaagcggcggcgggccagtcg	
3A-IP-D3	ggactagtgcgcggcggtttgccgggctgggggatagaagcggcggcgggccagtcg	
3A-CP-D3	ggactagtgcgcggcggtttgccgggctggggacaagaagcggcggcgggccagtcg	
3A-MP-D3	ggactagtgcgcggcggtttgccgggctggggcatagaagcggcggcgggccagtcg	
3A-NP-D3	ggactagtgcgcggcggtttgccgggctggggattagaagcggcggcgggccagtcg	
3A-TP-D3	ggactagtgcgcggcggtttgccgggctggggagtagaagcggcggcgggccagtcg	
P30-P2-D1	accctcaaccaaaagctaacggtaaaattattaaactcggccgccgatgcgctaaacgc	Replaced hydrophilic fragments with P30 and P2
P30-P2-D2	gcggcgggccattctaaatgactagcagatactttaggaaccctcaaccaaaagctaac	
P30-P2-D3	atttagaatttgcttttatatactggggggcagaagcggcggcgggccattctaaatg	
P30-P2-D4	ggactagtttcagttatacctataaatttagaatttgcttttatat	
5566AA-D1	As shown above	Replaced hydrophilic fragment at C-terminal with P30, $gp100^{44–59}$ or $HA^{307–319}$
P30-D2	aaccaaaagctaacggtaaaattattaaagggggcagaagcggcggcgggccagtcgaggtcgccgccgacg	
P30-D3	ggactagtttctaaatgactagcagatactttaggaaccctcaaccaaaagctaacggtaaaat	
gp-D2	ccattcgggatacagttggcggttccagggggcagaagcggcggcgggccagtcgaggtcgccgccgacgt	
gp-D3	ggactagtgtcgaggcgctgggcctcagtccattcgggatacagttggcgg	
HA-D2	gtgttctgtttgacgtacttggggccagaagcggcggcgggccagtcgaggtcgccgccgacgt	
HA-D3	ggactagtggtcgcgagcttcagagtgttctgtttgacgtacttggg	
